# Frontotemporal dementia: a systematic review of artificial intelligence approaches in differential diagnosis

**DOI:** 10.3389/fnagi.2025.1547727

**Published:** 2025-04-10

**Authors:** Serena Dattola, Augusto Ielo, Giuseppe Varone, Alberto Cacciola, Angelo Quartarone, Lilla Bonanno

**Affiliations:** ^1^IRCCS Centro Neurolesi Bonino-Pulejo, Messina, Italy; ^2^Brain Stimulation Mechanisms Laboratory, Division of Depression and Anxiety Disorders, McLean Hospital, Belmont, MA, United States; ^3^Department of Psychiatry, Harvard Medical School, Boston, MA, United States; ^4^Brain Mapping Lab, Department of Biomedical, Dental Sciences and Morphological and Functional Imaging, University of Messina, Messina, Italy

**Keywords:** frontotemporal dementia, machine learning, differential diagnosis, Alzheimer's disease, neuroimaging, deep learning, Support Vector Machines, convolutional neural networks

## Abstract

**Introduction:**

Frontotemporal dementia (FTD) is a neurodegenerative disorder characterized by progressive degeneration of the frontal and temporal lobes, leading to significant changes in personality, behavior, and language abilities. Early and accurate differential diagnosis between FTD, its subtypes, and other dementias, such as Alzheimer's disease (AD), is crucial for appropriate treatment planning and patient care. Machine learning (ML) techniques have shown promise in enhancing diagnostic accuracy by identifying complex patterns in clinical and neuroimaging data that are not easily discernible through conventional analysis.

**Methods:**

This systematic review, following PRISMA guidelines and registered in PROSPERO, aimed to assess the strengths and limitations of current ML models used in differentiating FTD from other neurological disorders. A comprehensive literature search from 2013 to 2024 identified 25 eligible studies involving 6,544 patients with dementia, including 2,984 with FTD, 3,437 with AD, 103 mild cognitive impairment (MCI) and 20 Parkinson's disease dementia or probable dementia with Lewy bodies (PDD/DLBPD).

**Results:**

The review found that Support Vector Machines (SVMs) were the most frequently used ML technique, often applied to neuroimaging and electrophysiological data. Deep learning methods, particularly convolutional neural networks (CNNs), have also been increasingly adopted, demonstrating high accuracy in distinguishing FTD from other dementias. The integration of multimodal data, including neuroimaging, EEG signals, and neuropsychological assessments, has been suggested to enhance diagnostic accuracy.

**Discussion:**

ML techniques showed strong potential for improving FTD diagnosis, but challenges like small sample sizes, class imbalance, and lack of standardization limit generalizability. Future research should prioritize the development of standardized protocols, larger datasets, and explainable AI techniques to facilitate the integration of ML-based tools into real-world clinical practice.

**Systematic review registration:**

https://www.crd.york.ac.uk/PROSPERO/view/CRD42024520902.

## 1 Introduction

Neurodegenerative dementias are an increasingly common cause of mortality and disability worldwide, especially among the elderly. Alzheimer's disease (AD) is the most common cause of dementia (Feigin et al., 2019). However, recent epidemiological studies and the refinement of clinical criteria have revealed that frontotemporal dementia (FTD) is also a widespread form (Nuytemans et al., 2024).

While the exact etiopathogenesis of this complex and multifaceted disorder is unknown, FTD has been linked to various genetic mutations. Nearly 40% of all FTD cases are familial, meaning they occur in families with a history of the disorder (Seelaar et al., 2010). Several genes have been implicated in FTD, including MAPT, GRN, and C9orf72. Mutations in these genes can lead to the abnormal accumulation of tau or TDP-43 proteins in neurons, which is thought to contribute to the disease process (Giunta et al., 2021; Kertesz and Munoz, 2004).

FTD differs from other types of dementia, such as vascular dementia and Lewy body dementia (DLB), in its clinical presentation and underlying pathology. FTD is characterized by the progressive degeneration of the frontal and temporal lobes, leading to profound changes in personality, behavior, language abilities, along with occasional physical symptoms, including tremor, rigidity, akinesia, etc. (Neary et al., 1998). In contrast, Vascular dementia arises from cerebrovascular damage, progressing stepwise with abrupt onset after strokes (Gorelick et al., 2016), while DLB features visual hallucinations, Parkinsonism (e.g., tremors, rigidity), and fluctuating cognition (Walker et al., 2015).

FTD also differs from AD, which is commonly mistaken for it. While AD is typically associated with prominent memory loss, FTD primarily manifests through significant changes in social and personal behavior, neglect of personal care, impaired judgment, and aphasia (Ratnavalli et al., 2002). In addition to these distinct symptom profiles, FTD and AD present different patterns of brain atrophy. AD is primarily characterized by medial temporal lobe atrophy, particularly affecting the hippocampus and entorhinal cortex, regions critical for memory processing (Gold et al., 2012). Conversely, FTD predominantly involves the frontal and anterior temporal lobes, with a more pronounced atrophy pattern in the orbitofrontal cortex, anterior cingulate, and insula, depending on the specific clinical variant (Rohrer, 2012; Yu et al., 2021). These distinct symptom profiles are crucial for accurate diagnosis and subject specific management of each dementia type.

Furthermore, FTD is frequently underdiagnosed due to clinical overlap with various psychiatric disorders. The early symptoms of FTD, such as personality changes, impulsivity, and apathy, often mimic conditions like bipolar disorder, schizophrenia, or major depressive disorder, leading to misdiagnosis and delays in appropriate treatment (Antonioni et al., 2023; Chaudhary and Duggal, 2014).

FTD encompasses several subtypes, each affecting different aspects of behavior or language abilities. These include behavioral variant FTD (bvFTD), semantic variant primary progressive aphasia (svPPA), and nonfluent/agrammatic variant PPA (nfvPPA) (Gorno-Tempini et al., 2011). The updated clinical diagnostic criteria for the bvFTD by Rascovsky and colleagues (Rascovsky et al., 2011) highlight behavioral and cognitive symptoms to better distinguish bvFTD from AD and other dementias. bvFTD is characterized by prominent changes in behavior, personality, and social conduct. Individuals with bvFTD may exhibit disinhibition, impulsivity, apathy, and loss of empathy (Williams et al., 2005). These changes can have a profound impact on personal and social relationships, often leading to challenges in maintaining employment and engaging in daily activities. Behavioral disturbances can be distressing for both the affected individuals and their caregivers, necessitating a multidisciplinary approach to care. svPPA, on the other hand, primarily affects language skills. Individuals with svPPA experience difficulties in understanding and using words, as well as a decline in semantic memory, which is the ability to recognize and understand the meaning of words and objects. This subtype often leads to profound communication challenges, affecting not only verbal expression but also written language and comprehension (Josephy-Hernandez et al., 2023). As a result, individuals with svPPA may struggle to convey their thoughts and feelings, impacting their ability to maintain social connections and participate in activities that require effective communication (Hodges and Patterson, 2007). In nfvPPA, patients have difficulty producing speech but can still understand language. NfvPPA can lead to frustration and social withdrawal as communication becomes increasingly challenging. The impact of nfvPPA extends beyond verbal communication, affecting daily activities that require coordination and motor skills (Gorno-Tempini et al., 2004).

Currently, there is no cure for FTD. In terms of treatment, pharmacological interventions for FTD remain limited, with symptomatic management focusing on the alleviation of behavioral and cognitive symptoms. For example, selective serotonin reuptake inhibitors (SSRIs) can help manage behavioral symptoms, while speech and language therapy can support those with language difficulties (Gorno-Tempini et al., 2011). Non-pharmacological approaches, including behavioral interventions, cognitive rehabilitation, and caregiver support, play a crucial role in enhancing quality of life for patients with FTD and their families. Advances in diagnostic criteria, genetic discoveries, and neuroimaging modalities have enhanced our understanding of FTD heterogeneity and facilitated early diagnosis and disease monitoring. Despite therapeutic challenges, ongoing research efforts hold promise for the development of targeted treatments to mitigate the impact of FTD on affected individuals and their families, and, ultimately, a cure for this disease.

Machine learning, a subset of Artificial Intelligence (AI), has made notable progress in recent years, enhancing clinical applications in the diagnosis, prognosis, and treatment of neurodegenerative disorders, including AD and behavioral variant such as bvFTD. Through the application of advanced mathematical models, ML enables algorithms to learn from training data and identify patterns in new datasets. In the investigation of AD and bvFTD, ML has been used extensively to extract relevant information from complex neuroimaging data, resulting in accurate and reliable diagnostic models. This progress has led to the development of robust diagnostic tools for these conditions (Habes et al., 2020). As a consequence, ML has gained significant attention in the medical field as a tool for improving diagnostic accuracy, personalizing treatment plans, and optimizing patient outcomes. Moreover, ML's ability to iteratively refine its performance and uncover hidden patterns within data highlights its potential to transform healthcare and advance precision medicine (Rajkomar et al., 2019).

Various neuroimaging techniques, when combined with ML approaches, have proven effective in the diagnosis of neurodegenerative diseases. Structural magnetic resonance imaging (MRI), which is able to capture morphometric brain features, combined with traditional ML algorithms such as logistic regression classifier, has demonstrated high accuracy in distinguishing bvFTD and AD, from healthy controls (HCs) (Bachli et al., 2020). In addition to MRI, electroencephalography (EEG) has shown promise as a complementary tool for differential diagnosis, particularly in revealing unique patterns of neural activity (Miltiadous et al., 2021). Researchers are also leveraging ML methods for pattern analysis to enhance the classification and diagnosis of FTD from multimodal data and feature extraction techniques (Ducharme, 2023). Although ML-based approaches using MRI have achieved high accuracy in distinguishing dementia patients from controls, a key limitation is the generalizability of these models across diverse populations and clinical settings (Rathore et al., 2017). Moreover, the complexity of image-derived features often impedes the seamless translation of these findings into routine clinical practice, highlighting the need for streamlined, interpretable solutions that bridge research advancements with real-world diagnostic workflows.

In particular, deep learning (DL), a subset of ML, offers solutions to some of the limitations associated with preprocessing raw data, allowing for the exploration of sample complexity to a greater extent. Recent research indicates that deep network architectures, comparable to traditional ML models, can effectively address the differential diagnosis of neurodegenerative diseases (Spasov et al., 2019; Basaia et al., 2019; Hu et al., 2021). DL models have shown good performance at mining MRI features by utilizing the extensive depth, width, and inter-layer connections of neural networks. This capability allows them to extract hierarchical features that represent different levels of abstraction in a data-driven manner. As a result, these models significantly improve the accuracy and robustness of diagnostic applications. However, one of the major concerns with AI tools used to assist clinicians in diagnosis, evaluation, and treatment planning is the lack of interpretability of current models. DL neural networks are often perceived as opaque, with their intricate data processing making it nearly impossible to figure out how they arrive at predictions, such as class probabilities. In response, the field of explainable AI (XAI) (Gunning et al., 2019; Guidotti et al., 2018) has introduced pioneering methods like Local Interpretable Model-agnostic Explanations (LIME) (Ribeiro et al., 2016) and SHapley Additive exPlanations (SHAP) (Lundberg and Lee, 2017), which offer clear, localized insights into the decision-making process of black-box models by attributing specific contributions of features to individual predictions, thereby enhancing transparency and enabling a deeper understanding of AI behavior.

The objective of this study is to systematically assess the strengths and limitations of current AI models used in the differential diagnosis of FTD. Notably, while AD has been extensively studied in the context of ML applications (Kishore and Goel, 2024; Shukla et al., 2023; Moorthy et al., 2023), FTD remains underexplored. Specifically, FTD presents unique diagnostic challenges due to its overlap with other neurological disorders and its variable clinical manifestations across subtypes. Through a comprehensive analysis, we sought to assess their performance relative to existing literature and identify opportunities for further refinement. By providing an overview of the current state of AI applications in this field, this work seeks to inform both the clinical and research communities, while highlighting research gaps and potential directions for future advancements.

## 2 Materials and methods

The literature search followed the Preferred Reporting Items for Systematic Reviews and Meta-Analyses (PRISMA) guidelines (Page et al., 2021). The systematic review is registered in the PROSPERO database with the identifier CRD42024520902.

### 2.1 Eligibility criteria

To ensure the selection of relevant and high-quality studies for this systematic review, both inclusion and exclusion criteria were established. Studies were eligible for inclusion if they met the following criteria: (1) studies applying ML techniques specifically for the differential diagnosis between FTD and other neurological disorders, without predefining which disorders were considered; (2) studies employing supervised, unsupervised, or semi-supervised learning methods; (3) studies using clinical data (e.g., neuroimaging, neuropsychological, or biomarker data) for differential diagnosis; (4) studies involving human participants; (5) original research articles published in peer-reviewed journals; (6) papers written in English; (7) studies published from 2013 to 2024. Review articles, meta-analyses, systematic reviews, conference abstracts, editorials, and case reports were excluded.

### 2.2 Sources of information and search methodology

The literature search was conducted across the PubMed, Web of Science, and Embase databases, covering studies from 2013 to 2024. The selected range was chosen to capture the most recent advancements in the field while ensuring the inclusion of studies that reflect contemporary methodologies, technologies, and evolving theoretical frameworks. Studies published prior to 2013 may not fully represent the current state of research or the significant developments in the area under review. By focusing on the past decade, we aim to incorporate the latest findings that are most relevant to current practices and emerging trends. Given the highly specialized nature of the topic and the inclusion of databases that do not use MeSH indexing, MeSH terms were not utilized. Instead, a keyword-based search approach was adopted, targeting the title, abstract, and, where available, keyword fields. The search terms used were: (“frontotemporal dementia” OR “frontotemporal degeneration” OR “frontotemporal neurodegeneration” in Title) AND (“machine learning” OR “deep learning” OR “neural networks” OR “classifier” in Title, Abstract, Keywords) OR (“differential diagnosis” in Title) AND (“frontotemporal dementia” OR “frontotemporal degeneration” OR “frontotemporal neurodegeneration” in Title) AND (“machine learning” OR “deep learning” OR “neural networks” OR “classifier” in Title, Abstract, Keywords).

### 2.3 Process of selection and data collection

**Figure 1:**
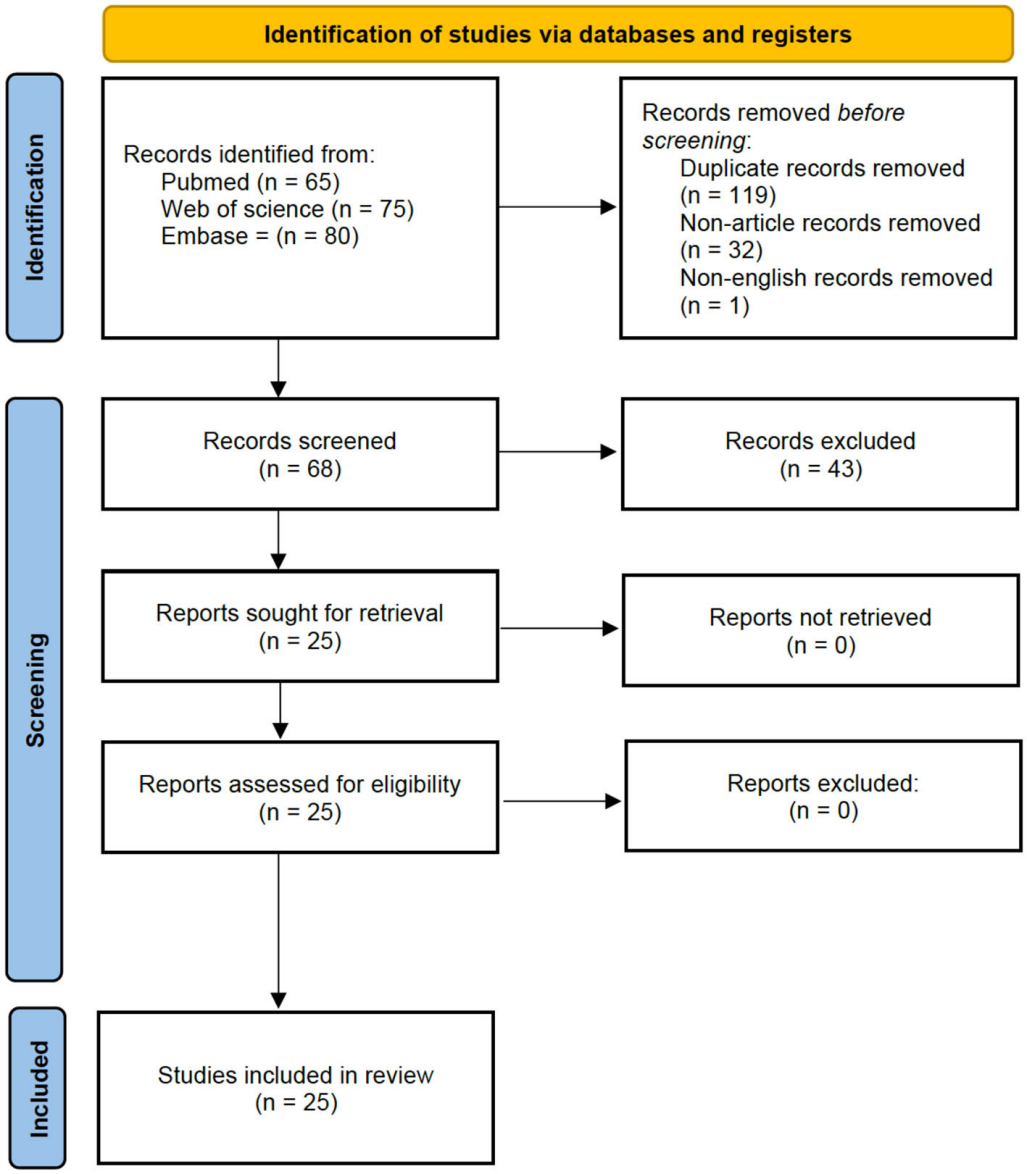
PRISMA flow diagram.

The study selection process was carried out in two phases. First, two independent reviewers screened the titles and abstracts of the records obtained from the search, applying the predefined inclusion and exclusion criteria, as reported in the previous section. In the second phase, the full texts of potentially eligible studies were independently reviewed by the same two reviewers for final inclusion. Figure 1 provides a PRISMA flow diagram that outlines the entire study selection process, including the number of studies screened, assessed for eligibility, and included, as well as reasons for exclusions at each stage. Data extraction from the included studies was performed using a customized spreadsheet that captured various study characteristics. Additionally, the two reviewers independently extracted information relevant to assessing the risk of bias in each study. Any differences that arose during the screening phase and data extraction between the two reviewers were resolved through discussion until a consensus was reached.

### 2.4 Data items

The following data were extracted from each study: 1) study details, including the first author and year of publication; 2) population characteristics, such as sample size, sub-groups, and age; 3) machine learning methods used; 4) key results and findings relevant to the review's focus; and 5) the outcomes of the risk of bias assessment.

### 2.5 Risk of bias assessment

The risk of bias for each study included in this systematic review was assessed using the National Institutes of Health (NIH) quality assessment tool for observational cohort and cross-sectional studies. Studies were rated as “Good,” “Fair,” or “Poor” based on their compliance with various criteria, such as study selection, comparability, exposure, and outcomes. These criteria included the clarity of the research question, the definition of the study population, the selection and measurement of exposure and outcomes, the consideration of potential confounders, the statistical analysis methods, and the reporting of participant recruitment and retention rates. Any differences in scoring between the reviewers were resolved through discussion until agreement was reached.

### 2.6 Outcome measures and data synthesis approaches

In this review, the performance of ML models for the differential diagnosis of FTD is mainly assessed using accuracy, which was the common evaluation measure among all the included studies. Additionally, the area under the receiver operating characteristic curve (AUC-ROC) is employed as a general measure of a model's ability to discriminate between FTD and non-FTD cases.

## 3 Results

The implemented search strategy identified 68 articles excluding duplicates, non-articles and non-English records. After reviewing the titles and abstracts, 25 studies met the eligibility criteria and were selected for full-text screening (Figure 1). All 25 studies were subsequently included in the review. Table 1 provides detailed information on the articles, including demographic data of participants, the aim of the study, information on the ML model, and findings.

**Table 1:** Summary table of the included studies.

**Study**	**Population (*n*)**	**Age (mean ±std)**	**Study aim**	**ML technique**	**Features**	**Findings**
Garcia-Gutierrez et al. (2022)	AD: *n* = 170; bvFTD: *n* = 72; HCs: *n* = 87	AD: 73.39 ± 8.13; bvFTD: 71.33 ± 7.75; HCs: 70.69 ± 8.59	To develop machine learning models for the diagnosis of AD and bvFTD using cognitive tests, and to differentiate between AD and bvFTD.	Evolutionary algorithms for feature selection and optimization; Classical machine learning algorithms (Naive Bayes, SVM, Decision Trees, RF, AdaBoost, and Gradient Boosting); Meta-model strategy for enhancing diagnosis accuracy.	Features were derived from cognitive scores adjusted by demographic factors. Evolutionary algorithms were applied to select significant features, reducing dimensionality while preserving diagnostic accuracy.	The study achieved high diagnostic accuracy levels (>84%) for AD, bvFTD, and differentiating between them.
Pérez-Millan et al. (2023)	AD: *n* = 153; FTD: *n* = 87; HCs: *n* = 99; 2-year follow-up data from 114 participants	Age at first MRI: AD: 64.3 ± 9.7; FTD: 63.6 ± 8.3; HCs: 60.2 ± 10.5; Age at second MRI: AD: 62.1 ± 4.5; FTD: 63.8 ± 5.9; HCs: 65.0 ± 7.2	To discriminate between AD and FTD using machine learning techniques applied to MRI data, both cross-sectionally and longitudinally.	PCA for feature reduction followed by SVM for classification.	Subcortical volumes and cortical thickness measures, transformed to z-scores after MRI processing.	SVM classifiers were applied to both cross-sectional and longitudinal MRI data, obtaining improved classification accuracy for FTD vs. HC in longitudinal analyses (87.8%), whereas distinguishing between AD and FTD in both analyses reached accuracies around 60%.
Garn et al. (2017)	Probable AD: *n* = 20; PDD or probable DLBPD: *n* = 20; bvFTD: *n* = 21	AD: 76.9 ± 6.7; PDD/DLBPD: 74.8 ± 8.5; bvFTD: 75.8 ± 5.7	To develop and evaluate a classifier for differentiating probable AD from PDD or DLB and from bvFTD based on QEEG.	SVM classifier, using feature reduction by Mann-Whitney U test and PCA.	25 QEEG features including relative band powers, spectral ratios, center frequency, auto-mutual information, cross-mutual information, coherences, phase coherences, partial coherences, Granger causality (GC), and conditional GC.	SVM classifiers achieved 100% accuracy in pairwise comparisons among AD, PDD or DLB, and FTD.
Wang et al. (2024)	AD: *n* = 36; FTD: *n* = 23; HCs: *n* = 29	AD: 66.4 ± 7.9; FTD: 63.7 ± 8.2; HCs: 67.9 ± 5.4	To investigate the potential of aperiodic components of EEG activity in distinguishing between AD and FTD.	SVM classifier, with oversampling using the SMOTE algorithm for sample imbalance and hyperparameters optimized with inner fivefold cross-validation.	Aperiodic parameters (offsets and exponents) and band-limited power of raw spectra (theta, alpha, and beta bands).	The combination of offsets, exponents, and the theta periodic power as features resulted in the best classification performance, with mean AUC = 0.73 ± 0.12.
Garćıa-Gutierrez et al. (2022)	AD: *n* = 171; bvFTD: *n* = 72; HCs: *n* = 87	NA	Development of a Python-based computational framework for early and automated diagnosis of AD and FTD using neuroimages and neurocognitive assessments.	Genetic Algorithms, specifically Mono-objective and Multi-objective Genetic Algorithms (NSGAII) for feature engineering, and a range of machine learning algorithms for predictive modeling (Bernoulli naive Bayes, SVM, KNN, Decision Trees, RF, AdaBoost, Gradient Boosting).	Features related to cognitive functions (memory, visuospatial, executive, attention, and language abilities) and brain metabolism data from FDG-PET analysis.	SVM binary classifiers obtained an accuracy of 92.6% in distinguishing between FTD, AD, and HC, combining PET imaging and cognitive data.
Nguyen et al. (2023)	Dataset ADNI2: AD: *n* = 149; HCs: *n* = 180; Dataset NIFD: FTD: *n* = 150; HCs: *n* = 136	ADNI2: AD: 74.7 ± 8.1; HCs: 73.4 ± 6.3; NIFD: FTD: 63.9 ± 7.1; HCs: 63.5 ± 7.4	To develop a deep learning-based framework for the differential diagnosis of AD and FTD using structural MRI data.	Deep Grading method incorporating U-Nets and an MLP.	Structure grading features and structure volume features.	The framework achieved an overall accuracy of 86.0% (87.9% on an external dataset) when distinguishing FTD, AD, and HCs. The accuracy for binary classification between FTD and AD was 94.6% (86.1% for the external dataset).
Birba et al. (2022)	AD: *n* = 33; bvFTD: *n* = 19; HCs: *n* = 42	AD: 74.65 ± 1.55; bvFTD: 68.57 ± 1.92; HCs: 69.87 ± 1.50	To assess whether bvFTD is characterized by an allostatic-interoceptive overload, evidenced by changes in rsHEP modulations, and its association with cognitive deficits and neuroimaging correlates.	Multimodal machine learning approach based on an XGBoost for classification.	rsHEP sources, functional connectivity features, and volumetric data from MRI.	Machine learning confirmed AIN specificity in predicting bvFTD. The classifier achieved an accuracy of 82% and an AUC of 0.81 in distinguishing bvFTD from AD.
Maito et al. (2023)	AD: *n* = 904; FTD: *n* = 282; HCs: *n* = 606	AD: 81.58 (9.95%); FTD: 72.33 (9.14%); HCs: 73.65 (10.9%)	Development of a novel framework for classification of AD, FTD, and HCs in multicentric heterogeneous samples from Latin American countries using unharmonized clinical, demographic, and cognitive assessments.	RF algorithm without bagging, non-linear SVM.	Measurements from cognitive screening, social cognition, neuropsychiatric symptoms, demographic variables.	The best model for discriminating between AD and FTD patients was the RF model (accuracy = 93.2%, AUC = 0.965).
Kim et al. (2019)	AD: *n* = 48; bvFTD: *n* = 48; svPPA: *n* = 50; nfvPPA: *n* = 39; HCs: *n* = 146	AD: 65.7 ± 7.6; FTD: 65.5 ± 11.8; HCs: 65.5 ± 15.0	To discriminate AD from FTD, and further, to determine the specific clinical syndrome within FTD, using MRI data.	Hierarchical classifier, single multi-label classifier.	Cortical thickness data.	The hierarchical classification framework achieved an accuracy of 90.8% in distinguishing FTD from AD, 86.9% between bvFTD and PPA, and 92.1% for nfvPPA vs svPPA.
Möller et al. (2016)	AD: *n* = 84; bvFTD: *n* = 51; HCs: *n* = 94	AD: 64.9 ± 7.1; bvFTD: 62.1 ± 7.8; HCs: 61 ± 7.3	To distinguish between AD and bvFTD in individual patients using MRI.	SVM.	Gray matter density maps.	SVM achieved accuracy of 82% for AD vs. bvFTD.
Bouts et al. (2018)	AD: *n* = 30; bvFTD: *n* = 23; HCs: *n* = 35	AD: 66.9 ± 7.8; bvFTD: 63.5 ± 7.6; HCs: 60.8 ± 6.1	To differentiate between AD and bvFTD on an individual basis using MRI, DTI, and rs-fMRI data.	Elastic net regression classifier.	Structural, DTI, and functional connectivity measures.	An accuracy of 77.7% (AUC = 0.81) for FTD vs. AD differentiation was achieved when combining mean diffusivity, full correlations between rs-fMRI-derived independent components, and fractional anisotropy.
Wang et al. (2016)	AD: *n* = 54; bvFTD: *n* = 55; HCs: *n* = 57	AD: 63.7 ± 8.1; bvFTD: 61.2 ± 9.4; HCs: 67.3 ± 6.8	To differentiate AD and bvFTD using MRI and neuropsychological data.	Naive Bayes.	MRI volumes, neuropsychological and neuropsychiatric measures.	Classification accuracies were 51.38% (MRI volumes), 62.39% (neuropsychological), and 61.47% (combined features) for distinguishing AD from bvFTD.
Lage et al. (2021)	AD: *n* = 18; bvFTD: *n* = 18; svPPA: *n* = 7; HCs: *n* = 29	AD: 68.17 ± 6.96; bvFTD: 68.83 ± 8.71; svPPA: 70.86 ± 8.11; HCs: 66.21 ± 5.51	To differentiate AD, bvFTD, and svPPA using oculomotor parameters.	SVM, KNN.	Eye movement parameters from various oculomotor tests.	KNN achieved an accuracy of 92.46% bvFTD vs. AD, outperforming SVM.
D́ıaz-Álvarez et al. (2022)	AD: *n* = 88; bvFTD: *n* = 81; PPA: *n* = 68; HCs: *n* = 39	AD: 73.90 ± 9.51; bvFTD: 70.68 ± 8.36; PPA: 72.62 ± 8.00; HCs: 68.06 ± 5.67	To diagnose AD and FTD using FDG-PET imaging combined with genetic algorithms for feature selection.	BayesNet Naive, KNN.	FDG-PET imaging data.	BayesNet Naive classifier achieved a high accuracy of 98.8% for FTD vs. AD, outperforming KNN.
Raamana et al. (2014)	Probable AD: *n* = 34; bvFTD: *n* = 30; HCs: *n* = 14	AD: 55.45 ± 3.06; bvFTD: 57.81 ± 3.36; HCs: 55.40 ± 4.72	To distinguish among AD, FTD, and HCs using MRI-based biomarkers.	Linear SVM, Non-linear SVM with Gaussian kernel, BayesNet classifier.	Volumes, Laplacian invariants, and surface displacements.	Using ventricular displacement features, the non-linear SVM model achieved a weighted AUC of 0.653 for AD vs. bvFTD.
Ajra et al. (2023)	AD: *n* = 36; FTD: *n* = 23; HCs: *n* = 29	AD: 66.4 ± 7.9; FTD: 63.6 ± 8.2; HCs: 67.9 ± 5.4	To improve diagnostic accuracy for dementia by classifying AD, FTD, and HCs groups using EEG signals.	Shallow CNNs, SVM, KNN, LDA.	Spectral-temporal features and functional connectivity measures.	Shallow CNN-based model achieved the highest accuracy (94.54%) with the AEC method in non-thresholded connectivity matrices.
Ma et al. (2020)	AD: *n* = 459; FTD: *n* = 434; HCs: *n* = 1063	AD: 75.91 ± 7.54; FTD: 64.69 ± 8.51; HCs: 72.19 ± 8.28	Differential diagnosis among AD, FTD, and HCs using multi-scale, multi-type MRI-based features with ensemble classifier and GAN strategy.	Multi-Scale Multi-Type Feature Deep Neural Network.	Volume size and cortical thickness.	MMDNN augmented with GANs achieved an accuracy of 88.28% in classifying FTD, AD, and HCs.
Hu et al. (2021)	Dataset ADNI: AD: *n* = 422; HCs: *n* = 469; Dataset NIFD: FTD: *n* = 552; HCs: *n* = 354	ADNI: AD: 75.5 ± 7.79; HCs: 75.3 ± 6.19; NIFD: FTD: 65.1 ± 7.48; HCs: 64.9 ± 7.85	To classify FTD, AD, and HCs using MRI data.	DL-based network.	Raw 3D MRI data.	The model achieved classification accuracies of 93.05% for distinguishing FTD from HCs, and 93.05% for differentiating FTD from AD.
Miltiadous et al. (2021)	AD: *n* = 10; FTD: *n* = 10; HCs: *n* = 8	AD: 70.5 ± 7.1; FTD: 67.5 ± 4.5; HCs: 68.5 ± 7.2	To compare six supervised machine learning techniques for classifying EEG signals of AD and FTD patients.	Decision Trees, RF, ANN, SVM, Naive Bayes, KNN.	Statistical and spectral features extracted from EEG signals.	Random Forests and Decision Trees showed the best performance for classifying FTD from AD (accuracy of 97.7% and 93.8%, respectively). SVM and KNN models also showed high performances (accuracies > 90%).
Rogeau et al. (2024)	AD: *n* = 199; FTD: *n* = 192; HCs: *n* = 200	AD: 68.4 ± 10.4; FTD: 68.4 ± 8.5; HCs: 71.6 ± 7.8	To introduce a 3D convolutional neural network for classifying participants as AD, FTD, or CN based on brain glucose metabolism using [18F]-FDG-PET scans.	3D CNN with VGG16-like architecture.	Brain FDG-PET volumes.	The model achieved an overall accuracy of 89.8% in distinguishing between FTD, AD, and HCs. In a complementary analysis with FTD and AD data only, model accuracy was 87.2%.
Lal et al. (2024)	AD: *n* = 36; FTD: *n* = 23; HCs: *n* = 29	AD: 66.4 ± 7.9; FTD: 63.7 ± 8.2; HCs: 67.9 ± 5.4	To evaluate and compare multiple feature extraction techniques and machine learning methods for discriminating between FTD, AD, and HCs using EEG data.	KNN, RF, XGBoost, and Extra Trees (ET).	Various feature extraction techniques from EEG signals, including SVD Entropy, HFD, ZCR, DFA, Hjorth Parameters.	The KNN classifier achieved an accuracy of 91% for FTD vs. AD using SVD entropy for EEG feature extraction and 90% overlap for sliding windowing.
Pérez-Millan et al. (2024)	AD: *n* = 215; FTD: *n* = 103; HCs: *n* = 173	AD: 65.0 ± 9.9; FTD: 63.7 ± 8.3; HCs: 59.4 ± 15.0	To develop a probabilistic computer-aided classification method for FTD and AD using MRI and CSF data, and to evaluate the improvement in diagnosis confidence by combining these modalities.	SVM classifier.	Cortical thickness (CTh) and gray matter volumes of different brain regions, CSF biomarkers.	Combining MRI-derived structural data with CSF biomarkers and age, SVM provided a classification accuracy between AD and FTD of 88.5%.
Sadeghi et al. (2024)	AD: *n* = 32; MCI: *n* = 103; FTD: *n* = 151; HCs: *n* = 147	AD: 73.4 ± 7.1; MCI: 71.2 ± 7.4; FTD: 64.5 ± 2.5; HCs: 67.6 ± 8.9	To develop a multimodal machine learning model that classifies AD, MCI, FTD, and HCs using features extracted from rs-fMRI and clinical data.	XGBoost.	Features extracted included temporal and spatial features from fMRI signals, such as absolute sum of changes, approximate entropy, Lempel-Ziv complexity, and Fourier coefficients.	The model using combined fMRI and clinical data achieved a balanced accuracy of 91.1% and a macro-averaged AUC of 0.99, significantly improving the classification of AD, FTD, mild MCI, and HCs compared to imaging data alone.
Ma et al. (2024)	bvFTD: *n* = 173; nfvPPA: *n* = 63; svPPA: *n* = 41	Overall: 63.7 ± 7.7	To differentiate patients with three clinical phenotypes of FTD (bvFTD, svPPA, and nfvPPA) using explainable deep learning models on structural MRI data.	MLP-based DNN classifier with multi-level parallel feature embedding.	Multi-type structural features from MRI scans: 360 cortical thickness features, 360 cortical volume features, and 15 subcortical volume features. Data were harmonized to control for confounders such as scanner differences and demographic variations using a GLM.	The DNN model achieved a balanced accuracy of 79.7% for bvFTD, 81.9% for nfvPPA, and 89.2% for svPPA. The overall balanced accuracy was 83.6%.
Rostamikia et al. (2024)	AD: *n* = 36; FTD: *n* = 23; HCs: *n* = 29	AD: 66.4 ± 7.9; FTD: 63.6 ± 8.2; HCs: 67.9 ± 5.4	To classify AD, FTD, and HCs using EEG signals and explore the most discriminative EEG features to differentiate AD from FTD.	SVM with Gaussian and linear kernels, KNN, Naive Bayes, and RF.	Basic time-domain features (mean, variance), frequency-domain features (subbands power), connectivity features (cross-correlation, coherence), and complex features (Katz fractal dimension, Lyapunov exponents, approximate entropy).	SVM model achieved an accuracy of 87.8% for differentiating between AD and FTD, and 93.5% for differentiating dementia (AD + FTD) from HCs.

AD, Alzheimer's Disease; ADNI2, Alzheimer's Disease Neuroimaging Initiative 2; AEC, Amplitude Envelope Correlation; AIN, Allostatic Interoceptive Network; ANN, Artificial Neural Network; AUC, Area Under the Curve; bvFTD, Behavioral Variant Frontotemporal Dementia; CNN, Convolutional Neural Network; CSF, Cerebrospinal Fluid; CTh, Cortical Thickness; DFA, Detrended Fluctuation Analysis; DLBPD, Dementia with Lewy Bodies/Parkinson's Disease; DNN, Deep Neural Network; DTI, Diffusion Tensor Imaging; EEG, Electroencephalography; ET, Extra Trees Classifier; FA, Fractional Anisotropy; FC, Functional Connectivity; FDG-PET, Fluorodeoxyglucose Positron Emission Tomography; FTD, Frontotemporal Dementia; GAN, Generative Adversarial Network; GLM, General Linear Model; GM, Gray Matter; HCs, Healthy Controls; HFD, Higuchi Fractal Dimension; KNN, k-Nearest Neighbors; MCI, Mild Cognitive Impairment; MLP, Multi-Layer Perceptron; MRI, Magnetic Resonance Imaging; NB, Naïve Bayes; NC, Normal Control; nfvPPA, Nonfluent Variant Primary Progressive Aphasia; NIFD, Neuroimaging Frontotemporal Dementia; NSGAII, Non-dominated Sorting Genetic Algorithm II; PCA, Principal Component Analysis; PDD, Parkinson's Disease Dementia; PET, Positron Emission Tomography; PPA, Primary Progressive Aphasia; RF, Random Forests; rs-fMRI, Resting-State Functional Magnetic Resonance Imaging; rsHEP, Resting-State Heartbeat Evoked Potential; SVD, Singular Value Decomposition; SVM, Support Vector Machine; SMOTE, Synthetic Minority Over-sampling Technique; svPPA, Semantic Variant Primary Progressive Aphasia; XGBoost, Extreme Gradient Boosting; ZCR, Zero Crossing Rate.

### 3.1 Risk of bias assessment

The included studies were evaluated using the 14-item NIH quality assessment tool for observational cohort and cross-sectional studies. The evaluation revealed a strong methodological framework, with all studies clearly articulating their objectives. Each study effectively assessed varying levels of exposure in relation to the outcomes and consistently applied well-defined, validated, and reliable exposure and outcome measures across participants. The overall quality of the studies was predominantly rated as “Good,” indicating that almost all demonstrated good methodological quality (Table 2). Only one study received a “Fair” rating due to the lack of demographic data of the participants involved, which impacts the study's transparency and limits its generalizability.

**Table 2:** Risk of bias assessment.

**Study**	**Item number**														**Quality rating**
	**1**	**2**	**3**	**4**	**5**	**6**	**7**	**8**	**9**	**10**	**11**	**12**	**13**	**14**	
Garcia-Gutierrez et al. (2022)	Yes	Yes	NA	Yes	No	NA	NA	NA	Yes	NA	Yes	NR	NA	Yes	Good
Pérez-Millan et al. (2023)	Yes	Yes	NA	Yes	No	NA	NA	NA	Yes	Yes	Yes	NR	No	Yes	Good
Garn et al. (2017)	Yes	Yes	NA	Yes	No	NA	NA	NA	Yes	NA	Yes	NR	NA	Yes	Good
Wang et al. (2024)	Yes	Yes	NA	Yes	No	NA	NA	NA	Yes	NA	Yes	NR	NA	Yes	Good
Garćıa-Gutierrez et al. (2022)	Yes	No	NA	Yes	No	NA	NA	NA	Yes	NA	Yes	NR	NA	Yes	Fair
Nguyen et al. (2023)	Yes	Yes	NA	Yes	No	NA	NA	NA	Yes	NA	Yes	NR	NA	Yes	Good
Birba et al. (2022)	Yes	Yes	NR	Yes	Yes	NA	NA	NA	Yes	NA	Yes	NR	NA	Yes	Good
Maito et al. (2023)	Yes	Yes	NR	Yes	No	NA	NA	NA	Yes	NA	Yes	NR	NA	Yes	Good
Kim et al. (2019)	Yes	Yes	NA	Yes	No	NA	NA	NA	Yes	NA	Yes	NR	NA	Yes	Good
Möller et al. (2016)	Yes	Yes	NA	Yes	No	NA	NA	NA	Yes	NA	Yes	NR	NA	Yes	Good
Bouts et al. (2018)	Yes	Yes	NA	Yes	No	NA	NA	NA	Yes	NA	Yes	NR	NA	Yes	Good
Wang et al. (2016)	Yes	Yes	NA	Yes	No	NA	NA	NA	Yes	NA	Yes	NR	NA	Yes	Good
Lage et al. (2021)	Yes	Yes	NA	Yes	No	NA	NA	Yes	Yes	NA	Yes	NR	NA	Yes	Good
D́ıaz-Álvarez et al. (2022)	Yes	Yes	NR	Yes	Yes	NA	NA	NA	Yes	NA	Yes	NR	NA	Yes	Good
Raamana et al. (2014)	Yes	Yes	NR	Yes	No	NA	NA	NA	Yes	NA	Yes	NR	NA	Yes	Good
Ajra et al. (2023)	Yes	Yes	NA	Yes	No	NA	NA	NA	Yes	NA	Yes	NR	NA	Yes	Good
Ma et al. (2020)	Yes	Yes	NA	Yes	Yes	NA	NA	NA	Yes	NA	Yes	NR	NA	Yes	Good
Hu et al. (2021)	Yes	Yes	NA	Yes	No	NA	NA	NA	Yes	NA	Yes	NR	NA	Yes	Good
Miltiadous et al. (2021)	Yes	Yes	NA	Yes	No	NA	NA	NA	Yes	NA	Yes	NR	NA	Yes	Good
Rogeau et al. (2024)	Yes	Yes	NA	Yes	No	NA	NA	NA	Yes	NA	Yes	NR	NA	Yes	Good
Lal et al. (2024)	Yes	Yes	NA	Yes	No	NA	NA	NA	Yes	No	Yes	NR	NA	Yes	Good
Pérez-Millan et al. (2024)	Yes	Yes	NR	Yes	No	Yes	Yes	Yes	Yes	No	Yes	NR	NA	Yes	Good
Sadeghi et al. (2024)	Yes	Yes	NA	Yes	No	NA	NA	NA	Yes	Yes	Yes	NR	NA	Yes	Good
Ma et al. (2024)	Yes	Yes	NA	Yes	No	Yes	NA	NA	Yes	No	Yes	NR	NA	Yes	Good
Rostamikia et al. (2024)	Yes	Yes	NA	Yes	No	NA	NA	NA	Yes	No	Yes	NR	NA	Yes	Good

NR, Not Reported; NA, Not Applicable. Items: 1 = Was the research question or objective in this paper clearly stated? 2 = Was the study population clearly specified and defined? 3 = Was the participation rate of eligible persons at least 50%? 4 = Were all the subjects selected or recruited from the same or similar populations (including the same time period)? Were inclusion and exclusion criteria for being in the study prespecified and applied uniformly to all participants? 5 = Was a sample size justification, power description, or variance and effect estimates provided? 6 = For the analyses in this paper, were the exposure(s) of interest measured prior to the outcome(s) being measured? 7 = Was the timeframe sufficient so that one could reasonably expect to see an association between exposure and outcome if it existed? 8 = For exposures that can vary in amount or level, did the study examine different levels of the exposure as related to the outcome (e.g., categories of exposure, or exposure measured as continuous variable)? 9 = Were the exposure measures (independent variables) clearly defined, valid, reliable, and implemented consistently across all study participants? 10 = Was the exposure(s) assessed more than once over time? 11 = Were the outcome measures (dependent variables) clearly defined, valid, reliable, and implemented consistently across all study participants? 12 = Were the outcome assessors blinded to the exposure status of participants? 13 = Was loss to follow-up after baseline 20% or less? 14 = Were key potential confounding variables measured and adjusted statistically for their impact on the relationship between exposure(s) and outcome(s)?

### 3.2 Samples, demographics and severity assessment

The review includes a total of 6,544 patients with dementia (mean age: 67.94 ± 5.24 years), involving 2,984 FTD (mean age: 66.18 ± 4.17 years), 3,437 AD (mean age: 69.5 ± 5.81 years), 103 mild cognitive impairment (MCI; mean age: 71.2 ± 7.4 years), 20 Parkinson's disease dementia or probable dementia with Lewy bodies (PDD/DLBPD; mean age: 74.8 ± 8.5 years). Mean age was calculated from the included studies reporting this information (24 out of 25). Most of the papers that met our inclusion criteria primarily focused on bvFTD, likely due to its higher prevalence in both clinical practice and research. To assess the severity status of participants with dementia across the reviewed articles, the most commonly used scales were the Mini-Mental State Examination (MMSE) and the Clinical Dementia Rating (CDR). These scales were mainly employed to evaluate the cognitive impairment in FTD, bvFTD, and AD. In four studies, other scales like the Montreal Cognitive Assessment (MoCA) and the Addenbrooke's Cognitive Examination (ACE) were also used.

### 3.3 Data types analyzed

To assess the patterns associated with FTD, AD, and other dementias, various neuroimaging, electrophysiological, cognitive, and behavioral data were analyzed across the included studies.

### 3.4 Machine learning techniques and models performance evaluation

SVMs, a widely used set of supervised ML algorithms, have emerged as the most frequently employed ML techniques in the reviewed studies (Miltiadous et al., 2021; Garcia-Gutierrez et al., 2022; Garćıa-Gutierrez et al., 2022; Pérez-Millan et al., 2023, 2024; Garn et al., 2017; Wang et al., 2024; Birba et al., 2022; Maito et al., 2023; Möller et al., 2016; Lage et al., 2021; Raamana et al., 2014; Rostamikia et al., 2024; Ajra et al., 2023; Ma et al., 2020). SVMs are favored for their robustness in handling high-dimensional data and their effectiveness in both binary and multi-class classification tasks. They have been applied to a diverse array of data types, including neuroimaging data such as structural MRI features (Pérez-Millan et al., 2023, 2024; Möller et al., 2016) and FDG-PET imaging (Garćıa-Gutierrez et al., 2022). Additionally, SVMs have been applied to EEG data (Garn et al., 2017; Wang et al., 2024; Rostamikia et al., 2024) and to cognitive and behavioral assessments (Garcia-Gutierrez et al., 2022; Maito et al., 2023).

In two studies, SVMs were combined with feature selection or dimensionality reduction techniques to enhance performance. Particularly, Principal Component Analysis (PCA) was employed to reduce feature dimensionality before classification (Pérez-Millan et al., 2023; Garn et al., 2017). These combinations aimed to improve the classifier's efficiency and accuracy by focusing on the most informative features.

Other ML algorithms were used across the reviewed literature. k-Nearest Neighbors (KNN), a straightforward non-parametric ML technique, was employed in seven studies (Miltiadous et al., 2021; Garćıa-Gutierrez et al., 2022; Lage et al., 2021; Rostamikia et al., 2024; Ajra et al., 2023; Lal et al., 2024; D́ıaz-Álvarez et al., 2022), often applied to cognitive, behavioral, and EEG data, sometimes in combination with feature selection methods. Several studies (Miltiadous et al., 2021; Garcia-Gutierrez et al., 2022; Garćıa-Gutierrez et al., 2022; Maito et al., 2023; Rostamikia et al., 2024; Lal et al., 2024) utilized Random Forests, employing ensemble learning to improve classification performance and robustness to overfitting. Other ensemble methods were used across studies: XGBoost (Birba et al., 2022; Lal et al., 2024; Sadeghi et al., 2024), AdaBoost (Garcia-Gutierrez et al., 2022; Garćıa-Gutierrez et al., 2022), Gradient Boosting (Garcia-Gutierrez et al., 2022; Garćıa-Gutierrez et al., 2022) and Extra Trees (Lal et al., 2024). Naive Bayes classifiers, a family of probabilistic algorithms, were applied in various studies (Miltiadous et al., 2021; Garcia-Gutierrez et al., 2022; Garćıa-Gutierrez et al., 2022; Rostamikia et al., 2024; D́ıaz-Álvarez et al., 2022; Wang et al., 2016). They proved effective in modeling of feature distributions, particularly with neuropsychological and imaging data. Elastic net regression was employed in one study (Bouts et al., 2018), offering a balance between feature selection and model complexity control. Linear discriminant analysis (LDA), a classification technique that maximizes class separability, was used in Kim et al. (2019) after applying a Laplace Beltrami operator and PCA, for noise removal and feature dimension reduction, respectively.

Deep learning models, including Convolutional Neural Networks (CNNs), were employed in six studies Hu et al. (2021); Ajra et al. (2023); Ma et al. (2020, 2024); Nguyen et al. (2023); Rogeau et al. (2024). These approaches use deep neural architectures to automatically identify patterns in raw data without requiring manual feature selection. For example, DL-based methods were applied to volumetric data derived from structural MRI (Hu et al., 2021; Ma et al., 2020, 2024; Nguyen et al., 2023). In Ma et al. (2020), the authors implemented a framework using Generative Adversarial Networks (GANs), a DL model designed to generate new data from an existing dataset, for data augmentation, combined with deep neural networks (DNNs) for classification. A 3D CNN was applied to FDG-PET scans (Rogeau et al., 2024) and shallow CNNs classified EEG-based spectral-temporal features and functional connectivity patterns, demonstrating versatility across data modalities (Ajra et al., 2023).

The performance of the ML models was primarily evaluated using metrics such as accuracy, sensitivity, specificity, and AUC-ROC. Accuracy measures the overall correctness of the model's predictions by calculating the proportion of correctly classified cases out of the total cases, and was reported in most studies. Sensitivity and specificity assess the model's ability to correctly identify true positives and true negatives, respectively. The AUC is a widely used metric for evaluating the discriminative ability of classifiers, with values closer to one indicating better performance. Some studies (Garcia-Gutierrez et al., 2022; Garćıa-Gutierrez et al., 2022; Maito et al., 2023; D́ıaz-Álvarez et al., 2022; Ajra et al., 2023; Lal et al., 2024; Pérez-Millan et al., 2024; Sadeghi et al., 2024) also employed metrics like F1-score, which balances sensitivity and precision by computing the harmonic mean of the two. The F1-score is especially valuable in datasets with class imbalance, ensuring that both false positives and false negatives are taken into account when evaluating model performance. K-fold, leave-one-out and nested cross-validation techniques were commonly used to assess model robustness and generalizability. These methods helped prevent overfitting and provided a more reliable estimate of model performance.

**Figure 2:**
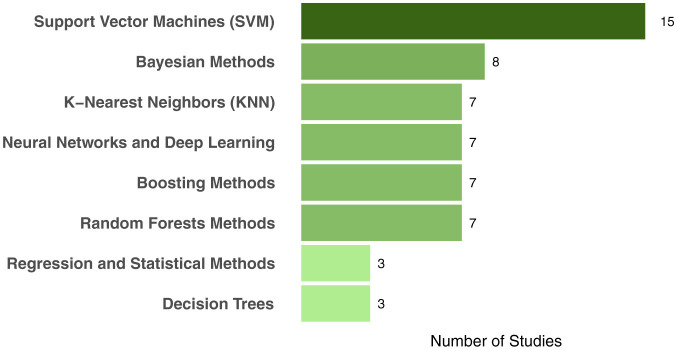
Frequency of ML approaches used across the included studies grouped by macro-categories.

Table 3, Figure 2 show the frequency of the ML approaches used across the included studies.

**Table 3:** Frequency of ML approaches used across the included studies.

**Machine learning approach**	**Frequency**	**Studies**
Support Vector Machines (SVM)	15	(Miltiadous et al., 2021; Garcia-Gutierrez et al., 2022; Garćıa-Gutierrez et al., 2022; Pérez-Millan et al., 2023, 2024; Garn et al., 2017; Wang et al., 2024; Birba et al., 2022; Maito et al., 2023; Möller et al., 2016; Lage et al., 2021; Raamana et al., 2014; Rostamikia et al., 2024; Ajra et al., 2023; Ma et al., 2020)
k-Nearest Neighbors (KNN)	7	(Miltiadous et al., 2021; Garćıa-Gutierrez et al., 2022; Lage et al., 2021; Rostamikia et al., 2024; Ajra et al., 2023; Lal et al., 2024; D́ıaz-Álvarez et al., 2022)
Naive Bayes	6	(Miltiadous et al., 2021; Garcia-Gutierrez et al., 2022; Garćıa-Gutierrez et al., 2022; Rostamikia et al., 2024; D́ıaz-Álvarez et al., 2022; Wang et al., 2016)
Random Forests	6	(Miltiadous et al., 2021; Garcia-Gutierrez et al., 2022; Garćıa-Gutierrez et al., 2022; Maito et al., 2023; Rostamikia et al., 2024; Lal et al., 2024)
Deep neural networks (DNNs)	4	(Hu et al., 2021; Ma et al., 2020, 2024; Nguyen et al., 2023)
Decision Trees	3	(Miltiadous et al., 2021; Garcia-Gutierrez et al., 2022; Garćıa-Gutierrez et al., 2022)
XGBoost	3	(Birba et al., 2022; Lal et al., 2024; Sadeghi et al., 2024)
Convolutional neural networks (CNNs)	2	(Ajra et al., 2023; Rogeau et al., 2024)
AdaBoost	2	(Garcia-Gutierrez et al., 2022; Garćıa-Gutierrez et al., 2022)
Gradient Boosting	2	(Garcia-Gutierrez et al., 2022; Garćıa-Gutierrez et al., 2022)
Linear discriminant analysis (LDA)	2	(Ajra et al., 2023; Kim et al., 2019)
Bayesian Networks	2	(Garcia-Gutierrez et al., 2022; Raamana et al., 2014)
Artificial Neural Networks (ANN)	1	(Miltiadous et al., 2021)
Elastic net regression	1	(Bouts et al., 2018)
Extra Trees (ET)	1	(Lal et al., 2024)

### 3.5 Models performances and findings

Many reviewed papers employed multiple ML methods simultaneously within their analyses, allowing for direct comparisons. In this section, we summarized the best-performing ML models for each included study, highlighting the most effective techniques contributing to improved diagnostic accuracy. The reviewed studies are grouped into sections based on the best-performing ML method employed.

#### 3.5.1 SVM

SVM classifiers have been extensively employed to enhance the differential diagnosis between FTD, AD, and HC. Overall, the accuracies reported for SVM-based methods ranged from approximately 60%–100%, with most studies achieving accuracies between 82% and 94.5%. This variation reflects differences in sample sizes, data modalities, and feature selection techniques, while demonstrating the strong performance of SVM classifiers in the differential diagnosis of neurodegenerative diseases. The included studies are organized into subsections, according to the type of data used for ML.

##### 3.5.1.1 Neuroimaging data

Structural MRI data analyzed using SVMs in Möller et al. (2016) resulted in accuracies of 85% for FTD vs. HC, and 82% for FTD vs. AD, demonstrating high predictive power across independent datasets. In Raamana et al. (2014), non-linear SVMs with Gaussian kernel were employed for direct three-way classification among AD, bvFTD, and normal controls. Using ventricular displacement features, the model achieved a weighted AUC of 0.765, with pairwise AUCs of 0.938 for bvFTD vs. HC, and 0.653 for bvFTD vs. AD, underscoring the diagnostic potential of ventricular morphology while also highlighting its limitations in distinguishing between dementia subtypes. In Pérez-Millan et al. (2023) the authors applied SVM classifiers to both cross-sectional and longitudinal MRI data, obtaining improved classification accuracy for FTD vs. HC in longitudinal analyses (87.8%), although distinguishing between AD and FTD remained challenging in both analyses with accuracies around 60%.

The potential of EEG features combined with SVMs was demonstrated in Garn et al. (2017), where classifiers achieved 100% accuracy in pairwise comparisons among groups of 20 AD, 20 PDD or dementia with Lewy bodies (DLB), and 21 FTD. The study underscores the efficacy of non-invasive EEG markers, although the authors highlighted the need for further studies using larger numbers of patients. In Wang et al. (2024), the integration of aperiodic EEG components of power spectral density with SVM classification significantly enhanced the differentiation between FTD and AD. The authors separated the raw power into periodic and aperiodic components, with the periodic components represented by periodic power and the aperiodic components represented by offsets and exponents. The study showed that using the combination of aperiodic parameters and the theta periodic power as features resulted in the best classification performance (mean AUC = 0.73 ± 0.12). In Rostamikia et al. (2024), the authors used SVM classifiers on EEG features, obtaining 93.5% accuracy for diagnosing dementia (FTD and AD vs. HC) and 87.8% for differentiating FTD from AD, underscoring the versatility of SVMs across different data types.

##### 3.5.1.2 Multimodal data

In Garcia-Gutierrez et al. (2022), SVM models utilizing neuropsychological assessments, adjusted by demographic factors, as features, achieved accuracies of 91.6% for FTD from HC, and 84.5% for differentiating AD from FTD. Furthermore, in Garćıa-Gutierrez et al. (2022), the authors showed that combining PET imaging and cognitive data using a multimodal ML approach including SVM binary classifiers, obtained an accuracy of 92.6% in distinguishing between FTD, AD and HC. Additionally, in Pérez-Millan et al. (2024), the integration of MRI-derived structural data, cerebrospinal fluid biomarkers, and age into SVM classifiers improved the classification accuracy between AD and FTD to 88.5%, providing a probabilistic assessment of diagnostic confidence. The accuracy of classification between FTD and HCs was 86.5%.

#### 3.5.2 DL methods

Deep learning methods, particularly CNNs, have also been effective in this domain. In Nguyen et al. (2023), a Deep Grading method incorporating U-Nets and a multi-layer perceptron (MLP) achieved an overall accuracy of 86.0% (87.9% on an external dataset) when distinguishing FTD, AD, and HC using MRI-based structural data. The accuracy for binary classification between FTD and AD was 94.6% (86.1% for the external validation dataset). Ajra et al. (2023) utilized a shallow CNN with four estimation methods of EEG-derived functional connectivity, achieving an accuracy of 94.54% in distinguishing from FTD, AD and HC when using the Amplitude Envelope Correlation (AEC) without any thresholding method. Similarly, in Ma et al. (2020), the authors developed a multi-scale, multi-type deep neural network (MMDNN) augmented with GANs, which achieved an accuracy of 88.28% in classifying FTD, AD, and HCs based on structural MRI features.

Hu et al. (2021) demonstrated the efficacy of deep learning applied to raw MRI data without any preprocessing or manual intervention by medical experts, achieving classification accuracies of 93.45% for distinguishing FTD from HCs, and 93.05% for differentiating FTD from AD. These findings underscore the potential of CNNs to autonomously capture intricate patterns within imaging data, highlighting their capability to obviate the need for extensive preprocessing and manual feature extraction in neuroimaging analyses. In Rogeau et al. (2024), a 3D CNN model using FDG-PET scans outperformed clinicians' interpretation in distinguishing between FTD, AD, and HCs, achieving an overall accuracy of 89.8%. In a complementary analysis with FTD and AD data only, model accuracy was 87.2%. Ma et al. (2024) introduced an explainable DNN that classified FTD subtypes with an overall balanced accuracy of 83.6%, providing insights into the structural markers specific to each subtype. To enhance model transparency and interpretability, they utilized an XAI technique called “Integrated Gradient”, which provides importance scores to each MRI input feature, highlighting their individual contributions to the model's predictions.

These DL studies reported accuracies ranging from 86.0% to 94.6%, indicating the potential of neural networks in employing complex neuroimaging and electrophysiological data for accurate diagnosis.

#### 3.5.3 Ensemble methods

Random Forests algorithms also demonstrated strong performance. In Maito et al. (2023), a Random Forests model achieved an accuracy of 93.2% and an AUC of 0.965 in differentiating FTD from AD using routine clinical and cognitive assessments in Latin American populations. Miltiadous et al. (2021) reported that Random Forests reached an accuracy of 97.7% for FTD vs. AD classification based on EEG features using a 10-fold cross-validation method.

Gradient boosting methods, such as XGBoost used in Sadeghi et al. (2024), combined fMRI time-course data with clinical and demographic variables (except for age) to achieve a balanced accuracy of 91.1%, significantly improving the classification of AD, FTD, mild cognitive impairment (MCI), and HCs compared to imaging data alone. A classifier based on an XGBoost was also used by Birba et al., who built a classifier algorithm using interoceptive EEG features, structural MRI measures, and functional connectivity markers to distinguish bvFTD from AD. The classifier achieved an accuracy of 82% and an AUC of 0.81, demonstrating the potential of integrating electrophysiological and neuroimaging biomarkers for reliable diagnosis Birba et al. (2022).

#### 3.5.4 Other ML methods

Naive Bayes classifiers were notably effective in certain studies. In Wang et al. (2016), the authors found that neuropsychological features analyzed with a Naive Bayes classifier achieved an accuracy of 62.39% in distinguishing AD from FTD, outperforming MRI-based measures (accuracy = 51.38). D́ıaz-Álvarez et al. (2022) used a BayesNet Naive classifier combined with genetic algorithm-based feature selection, achieving a high accuracy of 98.8% for FTD vs. AD using FDG-PET imaging.

KNN algorithm showed high efficacy in Lal et al. (2024), where it achieved an accuracy of 91% for FTD vs. AD, indicating its potential for EEG-based diagnostics. These results were achieved using SVD entropy for EEG features extraction, an XAI feature importance array, and 90% overlap for sliding windowing, In Lage et al. (2021), KNN was used on eye-tracking data achieving an accuracy of 92.46% for bvFTD vs. AD, outperforming SVM. KNN was also evaluated in D́ıaz-Álvarez et al. (2022), although it was outperformed by the BayesNet Naive classifier.

Other ML techniques were also employed. In Kim et al. (2019), LDA, a classical linear learning method, within a hierarchical classification framework achieved an accuracy of 90.8% in distinguishing FTD from AD, 86.9% between bvFTD and PPA, and 92.1% for nfvPPA vs svPPA. In Bouts et al. (2018) elastic net regression was used in a multiparametric model, achieving an accuracy of 77.7% (AUC = 0.81) for FTD vs. AD differentiation by integrating structural, diffusion tensor, and resting-state functional MRI measures.

### 3.6 Comparative analysis of ML techniques

A comparative analysis of ML techniques reveals distinct strengths and limitations in their application to the differential diagnosis of FTD (Table 4). Different models vary in terms of accuracy, interpretability, computational demands, and suitability for clinical implementation. DL models achieve high accuracy, particularly with neuroimaging data, but require large datasets and computational resources. In contrast, traditional ML methods such as SVMs, RF, and gradient boosting demonstrate strong performance with structured data like cognitive assessments and multimodal data. These models often require feature engineering but provide robust classification capabilities with more manageable computational demands. These variations highlight the importance of dataset composition, preprocessing, and feature selection in optimizing model performance.

**Table 4:** Comparative analysis of ML techniques.

**ML technique**	**Accuracy range**	**Pros**	**Cons**	**Common data modalities**
SVMs	60%–100%	Robust for high-dimensional data; good with small datasets	Limited interpretability; performance depends on feature selection	MRI, EEG, cognitive, multimodal
DL	86%–94.6%	Learns patterns automatically; excels with imaging data	Needs large datasets; high computational cost	MRI, EEG, FDG-PET
Random Forests	93.2%–97.7%	Handles missing data; interpretable; robust	Requires tuning; less effective for very high-dimensional data	EEG, cognitive, clinical
Gradient Boosting	82%–91.1%	High predictive power; effective with structured datasets	Prone to overfitting; computationally intensive	MRI, clinical, demographics
Naive Bayes	62.39%–98.8%	Fast, simple, good for small datasets	Assumes feature independence	FDG-PET, cognitive
KNN	91%–92.46%	Simple; good for small datasets	Sensitive to class imbalance; poor with high-dimensional data	EEG, Eye-tracking
LDA	86.9%–92.1%	Efficient, interpretable	Assumes linear separability; limited performance with complex data	MRI
Elastic net regression	77.7%	Reduces overfitting; effective for feature selection	Limited use in complex, non-linear problems; requires hyperparameter tuning	MRI

## 4 Discussion

This systematic review underscores the potential of ML techniques in improving the differential diagnosis of FTD, a critical challenge in clinical neurology.

Traditional diagnostic methods often rely on clinical assessments and neuropsychological tests, which may not capture subtle early-stage differences between these conditions (Bron et al., 2015; Vieira et al., 2017; Korolev et al., 2016). Early and accurate diagnosis and differentiation between FTD and other types of dementia, such as AD and PDD, are therefore essential for appropriate treatment planning and patient care. Indeed, different dementia types have distinct pathophysiological mechanisms and may respond differently to treatments.

By leveraging diverse data modalities such as neuroimaging and neuropsychological assessments, ML models offer a powerful tool for identifying subtle, disease-specific patterns that traditional methods may overlook. As it emerges from the revised papers, the performance of the ML algorithms often depends on the employed model and the data analyzed. Therefore the choice of the classification methodology can play a critical role in enhancing diagnostic performance across various types of dementia.

The ML techniques applied across the included studies are characterized by a predominance of SVMs and an increasing adoption of DL methods. SVMs have demonstrated consistent effectiveness in differentiating between FTD, AD, and HCs. The performance variation found across studies likely derives from heterogeneity in data sources, sample sizes, and features used.

While SVMs have remained a dominant choice due to their consistent effectiveness, recent years have seen a growing interest in DL models. These models, particularly those leveraging raw neuroimaging data, excel in identifying intricate patterns. This places them as competitive for multi-class tasks essential for real-world scenarios where clinicians need to differentiate among multiple neurodegenerative diseases. Notably, although several studies used DL approaches for multi-class classification, the highest accuracies are found in binary tasks (Nguyen et al., 2023; Hu et al., 2021).

Random Forests and Naive Bayes classifiers have demonstrated strong performance in binary classifications, with reported accuracies as high as 98.8% for FTD vs. AD, especially with neuroimaging data (D́ıaz-Álvarez et al., 2022; Miltiadous et al., 2021). However, these methods often lack the scalability and adaptability of DL models for complex datasets and were frequently outperformed by both DL techniques and SVMs. KNN algorithms also performed well, but one study reported that their efficacy is less robust than other approaches Garcia-Gutierrez et al. (2022); D́ıaz-Álvarez et al. (2022); Miltiadous et al. (2021); Rostamikia et al. (2024).

Regarding the features used by the ML studies analyzed in the review, integrating multimodal data, such as demographic, clinical, cognitive, structural and functional neuroimaging, and cerebrospinal fluid biomarker features, has been shown to improve diagnostic accuracy (Pérez-Millan et al., 2024; Bouts et al., 2018; Maito et al., 2023; Birba et al., 2022; Garćıa-Gutierrez et al., 2022; Sadeghi et al., 2024).

The application of ML approaches in the differential diagnosis of FTD from other types of dementia has shown great potential, particularly in the context of early diagnosis. Recent ML research in diagnosis has shifted from classifying a specific brain disease against controls to focusing on differential diagnosis. While earlier studies primarily relied on neuroimaging as a data source, current efforts emphasize the need of integrating multimodal data.

Although the reviewed studies demonstrate significant progress, several limitations exist. As reported in Table 1, many studies have small sample sizes, which may limit the generalizability of the models (Miltiadous et al., 2021; Garn et al., 2017; Wang et al., 2024; Birba et al., 2022; Lage et al., 2021; Raamana et al., 2014; Rostamikia et al., 2024; Ajra et al., 2023; Lal et al., 2024; Bouts et al., 2018). Class imbalance (Rahman and Davis, 2013) is another common issue found across the included articles. AD is consistently overrepresented compared to FTD and its subtypes, which can bias models toward better performance in AD classification. Recent research used GANs to address class imbalance in AD diagnosis. GANs have reconstructed missing PET images, improving classification performance on imbalanced datasets (Hu et al., 2021), and GAN-based oversampling methods have significantly enhanced brain disease diagnosis accuracy (Rezaei et al., 2020). Unsupervised GAN approaches detect AD at various stages by reconstructing adjacent brain MRI slices, achieving high diagnostic accuracy (Han et al., 2022). Additionally, GANs can generate synthetic brain MRI and PET images for different AD stages, addressing limited data in developing robust automated diagnosis models (Islam and Zhang, 2020). An interesting finding of our review is the prevalence of bvFTD in the examined sample. Further exploring different variants could provide more comprehensive insights into the differential diagnosis of FTD subtypes. Furthermore, the integration of longitudinal data could also improve the understanding of disease progression and enhance predictive modeling. Moreover, ethnic diversity represents a significant concern, as most studies rely on datasets from North America and Europe, with only a few incorporating data from Asia, Latin America, or other regions. This bias limits the generalizability of findings, potentially reducing diagnostic accuracy for diverse populations. Data source variability represents another challenge, particularly in MRI and PET-based studies, where different imaging protocols across centers can introduce inconsistencies. Although some papers implement harmonization techniques, many do not explicitly address these discrepancies, potentially impacting model performance.

In the context of clinical implementation, ML models must balance accuracy, interpretability, and computational efficiency. SVMs, RF, and LDA are the most clinically feasible due to their moderate computational requirements and interpretability, making them suitable for decision-support systems. Gradient boosting techniques, while effective, require careful tuning to prevent overfitting. Deep learning models, despite their accuracy, are challenging due to their black-box nature, high data demands, and computational cost, limiting their immediate integration into clinical practice. In this regard, the development of XAI techniques such as SHAP and LIME will be crucial for clinical adoption, as clinicians require transparency in decision-making processes. In our review, only two studies employed XAI techniques (Lal et al., 2024; Ma et al., 2024). XAI techniques can help elucidate how models make decisions, highlighting the most influential features and enabling clinicians to validate model outputs (Tjoa and Guan, 2020; Chaddad et al., 2023). XAI models can be broadly categorized into two approaches: model-agnostic (Jahan et al., 2023; Guan et al., 2022; Yousefzadeh et al., 2024) and model-specific (Umeda-Kameyama et al., 2021; Jahan et al., 2023). Model-agnostic methods, such as SHAP and LIME, provide general insights into model predictions by attributing outcomes to input feature contributions. Although SHAP quantifies feature importance, it remains in many respects a “black-box” method that often fails to highlight the interactions between features that drive model decisions (Al Olaimat et al., 2023; Brusini et al., 2024). In brain MRI analysis, for instance, SHAP identifies key features but does not always provide a clear, intuitive sense of how these features merge to shape predictions (Jahan et al., 2023). While model-agnostic techniques offer flexibility across diverse model types, model-specific methods focus on providing transparency and interpretability directly linked to individual AI models, particularly in DL and complex ML algorithms. Techniques like Layer-wise Relevance Propagation (LRP) (Bach et al., 2015) analyze contributions of individual neurons to final decisions, and Grad-CAM (Gradient-weighted Class Activation Mapping) (Selvaraju et al., 2017), visualizes areas of input that are important for predictions in CNN. These methods enhance the trust and effectiveness of AI systems by making their operations transparent and justifiable.

Additionally, a critical next step in advancing ML-based FTD diagnosis is real-world validation through prospective clinical trials. While many studies demonstrate high diagnostic accuracy using retrospective datasets, the true clinical applicability of these models remains uncertain without validation in real-world settings. Future studies should focus on integrating ML models into clinical workflows and testing their performance in prospective patient cohorts to ensure their robustness and clinical applicability. Moreover, prospective validation will enable clinicians to evaluate the practical challenges of implementing ML models in routine practice. Developing standardized protocols for real-world evaluation will be essential in bridging the gap between ML advancements and their clinical use for FTD diagnosis.

In summary, exploiting various data modalities and advanced algorithms, ML models can enhance diagnostic accuracy, leading to timely interventions and better patient outcomes. Addressing current limitations through standardization, larger datasets, and XAI will be essential for translating these advances into clinical practice.

## 5 Conclusions

This systematic review highlights significant advancements in applying ML techniques to differentiate FTD from other neurodegenerative conditions. To our knowledge, this is the first review to systematically evaluate machine learning algorithms specifically tailored to distinguish between FTD subtypes and to differentiate FTD from other neurodegenerative conditions, addressing a gap in the literature. The use of SVMs and deep learning algorithms, particularly CNNs, consistently achieved high diagnostic accuracy, showing particular promise in leveraging neuroimaging data for distinguishing FTD from AD and HCs. The integration of multimodal data, such as structural and functional neuroimaging, and neuropsychological assessments, has improved diagnostic performance by capturing complementary features across different domains of brain function. Despite these advancements, challenges such as small sample sizes, class imbalance, and lack of standardization limit the generalizability of current models. Future studies should prioritize creating and analyzing large, diverse, multi-center datasets to reduce bias and enhance generalizability. Standardized data collection protocols should focus on harmonizing imaging sequences, EEG preprocessing pipelines, and neuropsychological test administration to ensure consistency. Incorporating longitudinal data could provide insights into disease progression, enabling the development of models that predict both diagnosis and prognosis.

The development of XAI techniques could introduce transparency in ML models, increasing their interpretability and trustworthiness for clinical decision-making. To facilitate the transition from research to clinical practice, interdisciplinary collaboration between AI researchers, neurologists, and imaging experts is essential. Establishing standardized protocols and ensuring regulatory compliance will be crucial to successfully integrating ML-based diagnostics into routine healthcare. Additionally, ML-based medical tools must undergo rigorous regulatory approval processes, such as those required by the U.S. Food and Drug Administration (FDA) and European Conformity (CE) marking, to ensure safety, efficacy, and reliability in real-world clinical settings. Ethical considerations, including patient privacy, data security, and bias mitigation, should also be addressed to promote responsible AI application in neurology. Overall, ML approaches have shown promise in improving early and accurate diagnosis of FTD, potentially leading to timely interventions and better patient outcomes. Nevertheless, their success hinges on overcoming current challenges. Collaborative, interdisciplinary efforts combining methodological innovation with robust clinical validation will be essential for translating these advancements into the real-world applications.

## Data Availability

The original contributions presented in the study are included in the article, further inquiries can be directed to the corresponding author.
